# Economic evaluation of differentiated service delivery models for HIV treatment in Lesotho: costs to providers and patients

**DOI:** 10.1002/jia2.25692

**Published:** 2021-04-10

**Authors:** Brooke E Nichols, Refiloe Cele, Nkgomeleng Lekodeba, Betty Tukei, Nicoletta Ngorima‐Mabhena, Appolinaire Tiam, Thapelo Maotoe, Makatleho Veronica Sejana, Iyiola O Faturiyele, Charles Chasela, Sydney Rosen, Geoffrey Fatti

**Affiliations:** ^1^ Department of Global Health School of Public Health Boston University Boston MA USA; ^2^ Health Economics and Epidemiology Research Office Department of Internal Medicine School of Clinical Medicine Faculty of Health Sciences University of the Witwatersrand Johannesburg South Africa; ^3^ Right to Care Centurion South Africa; ^4^ EQUIP Lesotho Maseru Lesotho; ^5^ Kheth’Impilo AIDS Free Living Cape Town South Africa; ^6^ Elizabeth Glaser Pediatric AIDS Foundation USA; ^7^ Department of Epidemiology & Biostatistics School of Public Health University of the Witwatersrand Johannesburg South Africa; ^8^ USAID Washington DC USA; ^9^ Division of Epidemiology and Biostatistics Department of Global Health Faculty of Medicine and Health Sciences Stellenbosch University Cape Town South Africa

**Keywords:** health systems, differentiated care, LMIC, retention, treatment, economic evaluation

## Abstract

**Introduction:**

Lesotho, the country with the second‐highest HIV/AIDS prevalence (23.6%) in the world, has made considerable progress towards achieving the “95‐95‐95” UNAIDS targets, but recent success in improving treatment access to all known HIV positive individuals has severely strained existing healthcare infrastructure, financial and human resources. Lesotho also faces the challenge of a largely rural population who incur a significant time and financial burden to visit healthcare facilities. Using data from a cluster‐randomized non‐inferiority trial conducted between August 2017 and July 2019, we evaluated costs to providers and costs to patients of community‐based differentiated models of multi‐month delivery of antiretroviral therapy (ART) in Lesotho.

**Methods:**

The trial of multi‐month dispensing compared 12‐month retention in care among three arms: conventional care, which required quarterly facility visits and ART dispensation (3MF); three‐month community adherence groups (CAGs) (3MC) and six‐month community ART distribution (6MCD). We first estimated the average total annual cost of providing HIV care and treatment followed by the total cost per patient retained 12 months after entry for each arm, using resource utilization data from the trial and local unit costs. We then estimated the average annual cost to patients in each arm with self‐reported questionnaire data.

**Results:**

The average total annual cost of providing HIV care and treatment per patient was the highest in the 3MF arm ($122.28, standard deviation [SD] $23.91), followed by 3MC ($114.20, SD $23.03) and the 6MCD arm ($112.58, SD $21.44). Per patient retained in care, the average provider cost was $125.99 (SD $24.64) in the 3MF arm and 6% to 8% less for the other two arms ($118.38, SD $23.87 and $118.83, SD $22.63 for the 3MC and 6MCD respectively). There was a large reduction in patient costs for both differentiated service delivery arms: from $44.42 (SD $12.06) annually in the 3MF arm to $16.34 (SD $5.11) annually in the 3MC (63% reduction) and $18.77 (SD $8.31) annually in 6MCD arm (58% reduction).

**Conclusions:**

Community‐based, multi‐month models of ART in Lesotho are likely to produce small cost savings to treatment providers and large savings to patients in Lesotho. Patient cost savings may support long‐term adherence and retention in care.

## 1 INTRODUCTION

Lesotho, the country with the second‐highest HIV/AIDS prevalence (23.6%) in the world, has made considerable progress towards HIV epidemic control, but it still face a gap in starting and retaining HIV‐positive individuals on antiretroviral treatment (ART). While 86% of people living with HIV are estimated know their status, just 72% of these — 62% of all those with HIV – are on ART [1]. Further expansion of ART delivery is needed, but Lesotho, like other countries, faces both financial and programmatic constraints in scaling‐up services and retaining patients in care.

Differentiated service delivery models (DSD models) are intended to increase treatment access for patients and to reduce provider costs [2] by adjusting the location of service delivery, reducing the frequency of healthcare interactions, and making other changes to the delivery model. Examples of DSD models include community adherence groups (CAGs) and community‐based medication pickup points. While provider cost reductions are expected, the actual effect of DSD models on provider costs remains uncertain. A modelling study in Malawi suggested that annual HIV treatment costs among those enrolled in DSD models were 10% to 11% less than the total HIV treatment cost per patient enrolled in conventional care [3]. A study based on empirical data in Zambia, in contrast, found that costs for DSD models were generally slightly higher than costs for conventional care.{Nichols, 2021 #254} Further cost estimates based on actual service delivery experience are needed.

The reported effect of DSD models on patient costs is more consistent. The little available evidence suggests that DSD models for ART delivery reduce patients transport costs significantly. In Malawi, for example multi‐month scripting and fast‐track refills reduced patient travel costs by 67% [3]. DSD models may also reduce the time required for obtaining ART, and thus save patients both time and opportunity costs in the form of lost income and work productivity [4]. Patients seeking HIV care and treatment in lower and middle‐income countries are expected to lose between $9‐$70 in income per year, depending on the type of occupation, due to missed work [5, 6, 7]. Both time spent and transport costs are particularly high for rural patients [7]. Reducing these costs has the potential to improve patient retention in care and thus overall ART coverage.

From a cluster randomized trial of community‐based differentiated models of multi‐month ART delivery, we evaluated the impact of DSD models for ART delivery on costs to providers and costs to patients in Lesotho.

## 2 METHODS

### 2.1 Study setting

The cluster randomized non‐inferiority trial on which this economic evaluation is based assessed the effectiveness of DSD models for ART delivery for stable patients in Lesotho [8, 9]. Patients were defined as stable and eligible for the trial if they were (i) 18 years of age or older; (ii) on ART ≥ six months with no periods of defaulting on treatment since the last VL result (ART default defined as missing seven or more consecutive days of ART); (iii) on a first‐line ART regimen; and (iv) had no ARV drug substitutions since the last VL result < 1,000 copies/ml and plasma or dried‐blood spot VL < 1,000 copies/ml. Patients were excluded if they were pregnant or less than 12 months postpartum and breastfeeding mothers, had comorbidities, or had been diagnosed with a WHO clinical stage 3 or 4 condition within the past three months [10].

The trial compared clinical and economic outcomes among three study arms: (i) three‐month ART supply at facilities (3MF, control); (ii) three‐month ART supply through community adherence groups (CAGs) (3MC) and (iii) six‐month ART supply through community distribution points (CAD) (6MCD). Thirty clusters (healthcare facilities) were allocated randomly, with stratification by setting (urban or rural) and geographic location (Maseru, Mafeteng and Mohale’s Hoek districts). Participants were enrolled between August 2017 and April 2018, and follow‐up continued until July 2019. Details and primary outcomes of the trial are reported elsewhere (ClinicalTrials.gov Identifier: NCT03438370) [8, 10].

### 2.2 Study interventions

The standard of care at the time of the study called for three‐month, facility‐based refills (3MF arm. Patients in this arm visited the health facility every three months and received a three‐month supply of ART at a time. CAGs (3MC arm) consisted of a group of six to twelve participants who lived in a similar geographic location and attended the same healthcare facility. The groups met on a three‐monthly basis in the community at a venue of their choice. A single CAG member visited the facility to collect a three‐month supply of ART for all members of the CAG; annual clinic visits were required for all patients. For the 6MCD arm, study participants were dispensed a six‐month supply of ART at their first clinic visit. Six months later, they received a six‐month supply of ART in the community at a health outreach point within their village. The health outreach team included a driver, nursing assistant, nursing officer and other facility‐based administrative support staff. Patients in the 6MCD arm returned to the health facility every 12 months for their routine annual clinical consultation and viral load testing and received refills for alternating six‐month periods at the health outreach points.

### 2.3 Outcomes

For this economic evaluation, we calculated the average annual cost per patient treated and annual cost per patient retained from the provider perspective and the annual cost to access care from the patient perspective. Retention was defined on an intention‐to‐treat basis and equalled 1‐attrition for each model, where attrition included death (all‐cause) and loss to follow‐up (LTFU). LTFU was defined as not collecting ART medications for >90 days after the last missed scheduled collection date [11, 12]. Because viral load results were missing for a substantial subset of the population (27%), viral suppression was not used as the outcome for the economic evaluation.

### 2.4 Patient‐level resource utilization

For the provider cost analysis, all resources utilized by study participants between model entry and 12 months after model entry were collected from patient records. Resources captured included scheduled facility visits, unscheduled facility visits, CAG interactions (interactions in which group members met in a CAG for support or delivered/received ART), CAD interactions (interactions in which an individual received ART in the community distribution model), antiretroviral medications dispensed and viral load tests performed.

Patient records did not distinguish between CAG interactions, CAD interactions and scheduled facility visits, though they did distinguish between scheduled and unscheduled visits. Patients self‐reported the number of times they had a CAG interaction between facility visits. For our base‐case scenario, we therefore made the following assumptions about the types of interactions recorded. For conventional care (3MF) all interactions in the patient file were assumed to be scheduled facility visits. For three‐month CAGs (3MC), we took the number of self‐reported CAG interactions as the true number of CAG interactions and assumed that each patient also had one scheduled facility visit per year, as called for in the guidelines. For six‐month CAD patients (6MCD) whose records showed two or more facility visits in the 12‐month period, one facility visit was assumed to be a CAD interaction and one a scheduled facility visit, as called for in the guidelines. For 6MCD patients with just one facility visit recorded in the 12‐month period, that visit was assumed to be a scheduled facility visit. We also counted unscheduled facility visits for patients in each arm.

### 2.5 Unit cost estimates

Provider costs included medications, laboratory tests, clinic visits, CAG and CAD interactions, infrastructure, equipment, other fixed facility costs and other costs associated with delivering care through the DSD models, such as transportation. Provider costs in each arm were estimated from the provider’s perspective using micro‐costing methods [13, 14]. Details on costing methods for each type of resource are presented in supplementary file 1. All costs were collected in Lesotho Loti (LSL) and converted to US Dollars (USD) using the 2018 annual average exchange rate of LSL 13.25/USD (Central Bank of Lesotho). All costs are reported in 2018 USD. All equipment costs were annualized at a discount rate of 5% over their useful life years.

### 2.6 Patient cost data collection and analysis

Patient costs included transportation costs to and from facility visits and DSD interactions and opportunity costs of patients’ time. Data to assess patient‐level transport costs were collected from a convenience sub‐sample of enrolled patients from each arm through semi‐structured interviews at study enrolment and at 12 months of follow‐up. Through an eleven‐item questionnaire, patients were asked questions regarding the number of visits to the facility, travel expenses for attending facility visits, travel time and distances travelled. We note that ART is offered free‐of‐charge to patients in Lesotho; questionnaire respondents were not asked about any additional direct out‐of‐pocket medical costs for accessing care.

To estimate the opportunity cost related to time spent interacting with the health system, we used a proxy for lost wages based on Lesotho’s national average minimum wage 13]. We then multiplied Lesotho’s national average minimum daily wage ($7.10 or LSL 93.50) [15] by the number of reported facility visits. Based on interviews with DSD implementors, we assumed a full day of missed wages for a facility visit, which required both travel and waiting time, and a quarter of a day of missed wages for a DSD interaction, which required less travel and no waiting time.

### 2.7 Sensitivity analysis

Given that patient records did not distinguish between CAG interactions and facility visits, we conducted a sensitivity analysis of the number of CAG visits assumed. In the base case, we took the number of self‐reported CAG interactions as the true number of CAG interactions and reduced the number of facility visits to one per year, as called for in guidelines. We then assessed what the impact on cost per patient treated, cost per patient retained and cost to patient would be if there were instead two or three facility visits in the 12‐month follow‐up period, rather than just one.

### 2.8 Ethical considerations

The parent trial received ethical approval from the Lesotho National Health Research Ethics Committee (Ref: ID49‐2017) and the Chesapeake Institutional Review Board (Pro00022263) . Written informed consent was sought from participants prior to conducting interviews.

## 3 RESULTS

A total of 5,336 participants enrolled in the trial, including 1,898 in the 3MF arm, 1,558 in the 3MC arm, and 1,880 in the 6MCD arm [10]. The median age was 42.7 (IQR 34.7 to 54.0) in the 3MF arm, 48.4 (IQR 39.4 to 57.8) in the 3MC arm, and 41.2 (IQR 33.3 to 51.4) in the 6MCD arm. Across all arms, the majority of participants were female: 1158 (61%) in the 3MF arm, 1117 (72%) in the 3MC arm and 1264 (67%) in the 6MCD arm. Approximately a quarter of participants lived greater than a 9km radius from their health facility (21.6% to 27.8%) [10]. The retention rates 12 months after enrolment were identical among study arms: 97.1% in 3MF (96.6% for women, 97.7% for men), 96.5% in 3MC (96.8% for women, 95.9% for men) and 94.7% in 6MCD (94.4% for women, 95.3% for men) [10].

### 3.1 Unit costs

**Figure 1:**
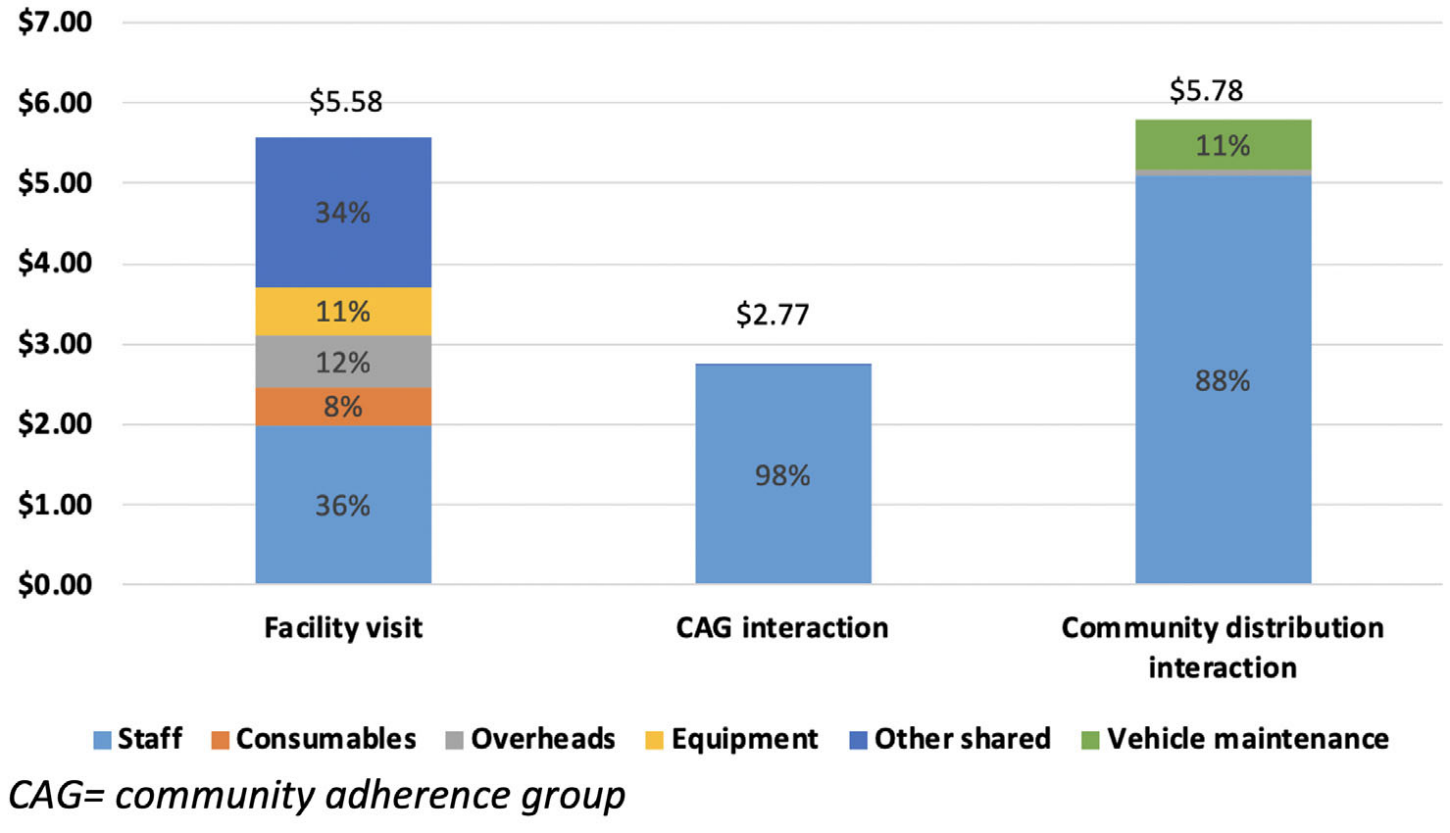
Unit cost of facility visit and differentiated service delivery interactions, by component. CAG, community adherence group.

The cost of a single facility visit was estimated to be $5.58, comprised primarily of staff costs (36%) and shared costs (34%) (Figure 1). A CAG interaction was estimated to cost $2.77, driven almost entirely by staff oversight costs (98%). The cost of a CAD interaction was similar to the cost of a facility visit at $5.78 per interaction, comprised primarily of staff costs (88%) and vehicle maintenance (11%).

### 3.2 Resource utilization

As expected, the 3MF arm had the highest mean number of facility visits per year (4.19), compared to 1.0 annual facility visits in the 3MC arm, and 1.49 annual facility visits in the 6MCD arm (Table 1). The highest number of unscheduled facility visits occurred in the 3MF arm (mean of 0.46/year), followed by 6MCD (0.23) and 3MC (0.04). The number of annual viral load tests conducted and number of ART days dispensed were roughly similar across arms.

**Table 1:** Resource use by arm

Resource	Conventional care (3‐monthly ART refill pick‐up, 3MF)	3‐month CAGs (3MC)	6‐month community distribution (6MCD)
	n = 1898	n = 1558	n = 1880
	Mean 2018 USD (SD)		
Facility visits	4.19 (0.70)	1.00 (0)^a^	1.49 (0.56)^b^
Unscheduled facility visits	0.46 (1.05)	0.04 (0.23)	0.23 (0.65)
CAG interactions	0	4.65 (2.44)	0
Community distribution point interactions	0	0	0.96 (0.18)^b^
Viral load	0.53 (0.56)	0.39 (0.52)	0.46 (0.53)
ART days dispensed	343 (56)	350 (59)	354 (52)

ART, antiretroviral treatment; CAG, community adherence group.

^a^ Assumed just one facility visit per patient

^b^ with any 6MCD patient that had >1 facility visit, assumed 1 community distribution visit; for patients with 0 or 1 visit recorded during the follow‐up period, assumed 0 community distribution visits.

### 3.3 Total cost of treatment and cost per patient retained

The average total annual cost of providing HIV care and treatment per patient was highest in the 3MF arm ($122.28, SD $23.91), followed by the 3MC arm ($114.20, SD $23.03) and the 6MCD arm ($112.58, SD $21.44). The provider cost per patient retained in care was approximately 6% lower for both the 3MC and 6MCD arms ($118.38 [SD $23.87] and $118.83 [SD $22.63] respectively) than for the 3MF arm ($125.99, SD $24.64) (Table 2).

**Table 2:** Provider costs and cost per person retained at 12‐month post model entry in each arm

Annual provider costs	Arm		
	Conventional care (3‐monthly ART refill pick‐up, 3MF)	3‐month CAGs (3MC)	6‐month community distribution (6MCD)
	Mean 2018 USD (SD)		
Facility visits	$23.36 ($3.89)	$5.58 (0)	$8.31 ($3.15)
Unscheduled facility visits	$2.55 ($5.84)	$0.20 ($1.27)	$1.29 ($3.61)
CAG interactions	–	$13.59 ($7.95)	–
Community pick‐up point interactions	–	–	$5.59 ($1.02)
Viral load	$12.00 ($12.66)	$8.93 ($11.75)	$10.29 ($11.97)
ART medication	$84.37 ($13.88)	$86.10 ($14.47)	$87.08 ($12.84)
Total cost (provider only)	$122.28 ($23.91)	$114.20 ($23.03)	$112.58 ($21.44)
Percent change (compared to 3‐month pick‐up)	Ref	−6.6%	−7.9%
Annual provider cost per person retained 12‐month post model entry	$125.99 ($24.64)	$118.38 ($23.87)	$118.83 ($22.63)
Percent change (compared to 3‐month pick‐up)	Ref	−6.0%	−5.7%

ART, antiretroviral treatment; CAG, community adherence group.

Total cost/patient was marginally higher for female than for male patients. Costs were $121.33 [SD $25.11] and $123.75 [SD $21.83], respectively, for women and men, respectively, in 3MF; $114.43 [SD $22.79] and $114.34 [SD $23.64] in 3MC; and $112.85 [SD $21.72] and $111.42 [SD $20.81] in 6MCD (Tables 3 and 4). The cost savings to the provider as measured by cost per patient retained in care was slightly higher for men (7.2% and 7.6% of annual costs saved for 3MC and 6MCD compared to 3MF respectively) than for women (5.3% and 4.6% of annual costs saved for 3MC and 6MCD compared to 3MF).

**Table 3:** Provider costs and cost per person retained at 12‐month post model entry in each arm‐ women

Annual provider costs	Arm		
	Conventional care (3‐monthly ART refill pick‐up, 3MF)	3‐month CAGs (3MC)	6‐month community distribution (6MCD)
	Mean 2018 USD (SD)		
Facility visits	$23.20 ($4.10)	$5.58 (0)	$8.23 ($3.15)
Unscheduled facility visits	$2.45 ($5.73)	$0.19 ($1.26)	$1.23 ($3.40)
CAG interactions	–	$13.32 ($7.82)	–
Community pick‐up point interactions	–	–	$5.59 ($1.02)
Viral load	$11.99 ($12.51)	$9.39 ($11.94)	$10.68 ($11.92)
ART medication	$83.69 ($14.68)	$85.95 ($14.47)	$87.12 ($13.11)
Total cost (provider only)	$121.33 ($25.11)	$114.43 ($22.79)	$112.85 ($21.72)
Percent change (compared to 3‐month pick‐up)	Ref	−5.7%	−7.0%
Annual provider cost per person retained 12‐month post model entry	$125.02 ($25.88)	$118.43 ($23.63)	$119.32 ($22.93)
Percent change (compared to 3‐month pick‐up)	Ref	−5.3%	−4.6%

ART, antiretroviral treatment; CAG, community adherence group.

**Table 4:** Provider costs and cost per person retained at 12‐month post model entry in each arm‐ men

Annual provider costs	Arm		
	Conventional care (3‐monthly ART refill pick‐up, 3MF)	3‐month CAGs (3MC)	6‐month community distribution (6MCD)
	Mean 2018 USD (SD)		
Facility visits	$23.61 ($3.52)	$5.58 (0)	$7.89 ($3.11)
Unscheduled facility visits	$2.71 ($6.01)	$0.24 ($1.27)	$1.43 ($4.00)
CAG interactions	–	$14.31 ($8.22)	–
Community pick‐up point interactions	–	–	$5.59 ($1.02)
Viral load	$12.01 ($12.89)	$7.74 ($11.17)	$9.51 ($12.03)
ART medication	$85.42 ($12.47)	$86.47 ($15.13)	$87.00 ($12.28)
Total cost (Provider only)	$123.75 ($21.83)	$114.34 ($23.64)	$111.42 ($20.81)
Percent change (compared to 3‐month pick‐up)	Ref	−7.6%	−10.0%
Annual provider cost per person retained 12‐month post model entry	$127.51 ($22.50)	$118.27 ($24.51)	$117.81 ($21.97)
Percent change (compared to 3‐month pick‐up)	Ref	−7.2%	−7.6%

ART, antiretroviral treatment; CAG, community adherence group.

### 3.4 Costs to patients

The annual cost to patients of obtaining ART was $44.42 (SD $12.06) in the 3MF arm, $16.34 (SD $5.11) in the 3MC arm, and $18.77 (SD $8.31) in the 6MCD arm (Table 5). In the 3MF arm, patient costs were driven by the need to take an entire day to visit a healthcare facility, compared to just a fraction of a day required for a DSD interaction in the community. Costs to patient did not vary by sex (Tables 6 and 7).

**Table 5:** Annual patient costs by arm

Annual patient costs	Arm		
	Conventional care (3‐monthly ART refill pick‐up, 3MF)	3‐month CAGs (3MC)	6‐month community distribution (6MCD)
	Mean 2018 USD (SD)		
Cost of transport	$11.45 ($3.03)	2.62 ($0.13)	$4.83 ($2.31)
Opportunity cost facility visits	$32.97 ($9.06)	$7.1 (0)	$12.22 ($5.96)
Opportunity cost CAG interactions	–	$6.62 ($5.09)	–
Opportunity cost community pick‐up point interactions	–	–	$1.72 ($0.31)
Total cost (Patient only)	$44.42 ($12.06)	$16.34 ($5.11)	$18.77 ($8.31)
Percent change (compared to 3‐month pick‐up)	Ref	−63%	−58%

CAG, community adherence group.

**Table 6:** Annual patient costs by arm‐ women

Annual patient costs	Arm		
	Conventional care (3‐monthly ART refill pick‐up, 3MF)	3‐month CAGs (3MC)	6‐month community distribution (6MCD)
	Mean 2018 USD (SD)		
Cost of transport	$11.37 ($3.05)	2.61 ($0.13)	$4.91 ($2.22)
Opportunity cost facility visits	$32.64 ($9.07)	$7.1 (0)	$12.28 ($5.68)
Opportunity cost CAG interactions	–	$6.45 ($5.01)	–
Opportunity cost community pick‐up point interactions	–	–	$1.72 ($0.31)
Total cost (Patient only)	$44.00 ($12.08)	$16.16 ($5.02)	$18.90 ($7.94)
Percent change (compared to 3‐month pick‐up)	Ref	−63%	−57%

CAG, community adherence group.

**Table 7:** Annual patient costs by arm‐ men

Annual patient costs	Arm		
	Conventional care (3‐monthly ART refill pick‐up, 3MF)	3‐month CAGs (3MC)	6‐month community distribution (6MCD)
	Mean 2018 USD (SD)		
Cost of transport	$11.57 ($3.01)	2.63 ($0.13)	$4.68 ($2.48)
Opportunity cost facility visits	$33.49 ($9.02)	$7.1 (0)	$12.10 ($6.50)
Opportunity cost CAG interactions	–	$7.07 ($5.27)	–
Opportunity cost community pick‐up point interactions	–	–	$1.71 ($0.31)
Total cost (Patient only)	$45.07 ($12.01)	$16.79 ($5.29)	$18.50 ($9.03)
Percent change (compared to 3‐month pick‐up)	Ref	−62%	−58%

CAG, community adherence group.

### 3.5 Sensitivity analysis

**Figure 2:**
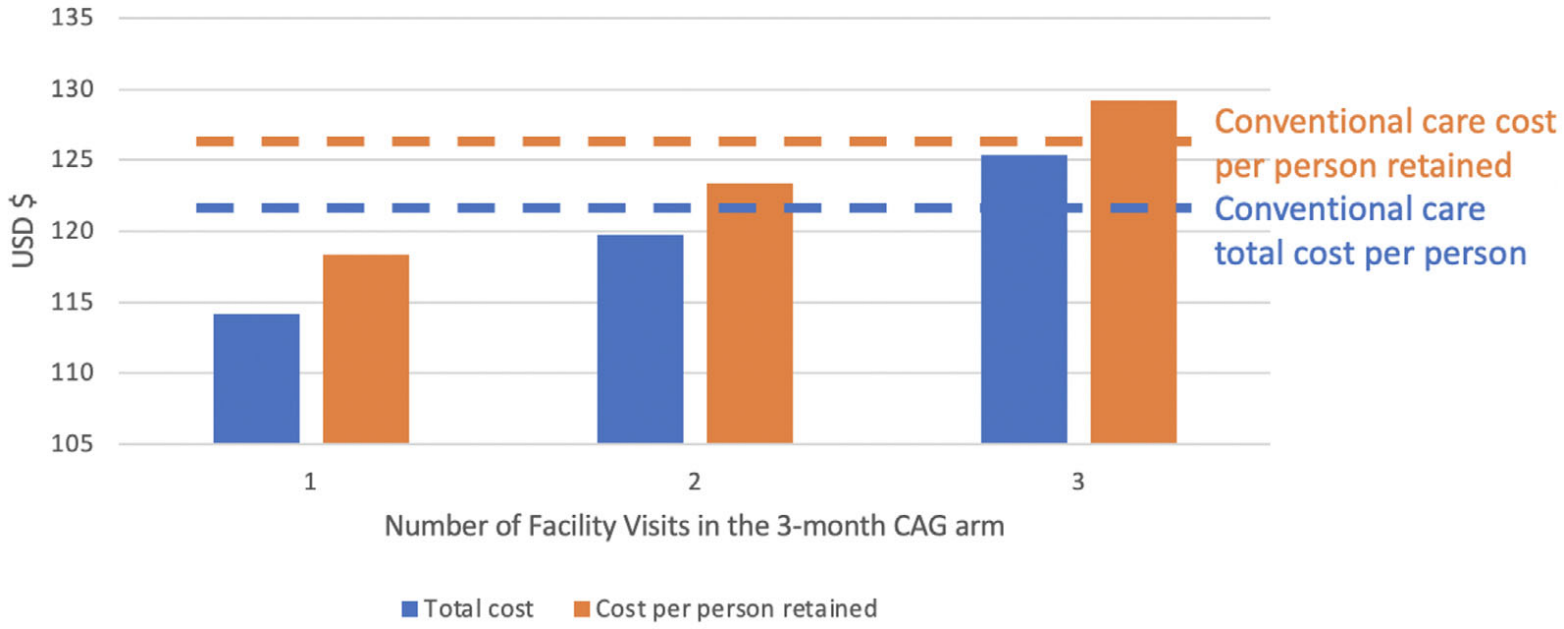
Change in number of facility visits assumed in the 3‐month CAG (community adherence group) arm (3MC) and provider costs.

As illustrated in Figure 2, increasing the number of facility visits in the 3MC arm increased the total annual cost per patient and per patient retained. Even assuming, however, an average of three facility visits per year, instead of the single annual facility visit assumed in the base case, the total cost per patient/per patient retained was only about $3 more per year than in the 3MF arm. This scenario of additional facility visits did affect patient costs more substantially (Table 8), though some net savings to patients persisted.

**Table 8:** Change in number of facility visits assumed in the three‐month CAG (3MC) arm and patient costs

	Number of facility visits assumed		
	1	2	3
Cost of transport	$2.62	$5.24	$7.86
Opportunity cost facility visits	$7.10	$14.20	$21.30
Opportunity cost CAG interactions	$6.62	$6.62	$6.62
Total cost (Patient only)	$16.34	$26.06	$35.78
Percent change (compared to conventional care, 3MF)	−63%	−41%	−20%

CAG, community adherence group.

## 4 DISCUSSION

In this economic evaluation of a cluster‐randomized trial of three‐ and six‐month dispensing of ART in Lesotho, we observed a small reduction in cost to the provider for both the 3MC and 6MCD models compared to conventional care (3MF) and substantial savings to patients for both DSD models.

Not surprisingly, costs for each model reflected the details of how the model was designed and implemented. For this trial in Lesotho, the cost of a community distribution interaction was similar to the cost of a facility visit due to the large number of staff involved in providing a community distribution point, which typically served only a small number of patients per day. Even so, the CAD model cost less than conventional care per patient served, because of the small number of annual interactions it required. Because details like the exact staff cadres utilized to implement a DSD model may determine whether it costs more or less than conventional care, country‐specific research on DSD model costs will likely remain an important contribution to HIV programme management.

Our main result of a 6% decrease in provider costs for the 6MCD model is roughly consistent with the modelled estimate made by Prust et al in Malawi using a guidelines‐based costing approach [3]. In Zambia, however, community‐based models of care were found to be more expensive than conventional care, as mentioned earlier [4]. The observed differences—higher costs in Zambia versus lower costs in Lesotho—may reflect differences in implementation details, as noted above, but they likely also reflect a true reduction in patients’ facility visits in Lesotho, which was not observed in Zambia. The large reduction in costs to the patient that we observed in Lesotho—savings of 63% and 58% in the 3MC and 6MCD models, respectively, is similar to what has been observed across a number of studies in several countries in sub‐Saharan Africa [3, 16, 17].

There were several limitations to our economic evaluation. First, we were unsure of the exact breakdown of DSD interactions versus facility visits for our DSD models. We addressed this through assumptions about likely number of DSD interactions given the expected implementation of the interventions as our base case and through variations in these assumptions in the sensitivity analysis. When the number of assumed facility visits increased for the CAG arm, the total cost and cost to patient increased, as expected. Second, we did not include programmatic costs of taking a DSD model to scale. While we found that the 3MC and 6MCD models cost roughly equivalent amounts to standard of care at the facility level, per patient treated, there were also likely costs incurred that we did not capture, such as those related to program scale‐up, oversight, and monitoring. If these costs were included, the cost of implementing these DSD models may be greater than the cost of implementing conventional care in the short term, though probably not over time. Third, patient transport costs were derived from a sub‐sample of the original cohort. The subsample surveyed in the 3MF and 3MC arms was more likely to live in urban settings than the original cohort, leading us to potentially underestimate average travel cost and time. Total costs to patients may also have been underestimated due to the exclusion of other personal expenditures, such as paid childcare or food.

## 5 CONCLUSIONS

Despite the limitations described above, our study is the first to provide empirical evidence on the impact of DSD models from both the provider and patient perspective in Lesotho. We expect small cost savings to providers of three‐month CAGs and six‐month community distribution of ARVs, compared to conventional care. We expect a substantial reduction in costs to patients, which may have significant benefits to the patient and support long‐term adherence and retention in care. Scaling up implementation of community‐based, multi‐month dispensing models may also be particularly important in the context of the COVID‐19 pandemic, both to decongest health facilities and minimize the number of interactions needed between patients and providers. With this in mind, policy makers should take into account public health benefits as well as savings to providers and patients in making decisions about scaling up these multi‐month dispensing models.

## COMPETING INTERESTS

We declare no competing interests.

## AUTHOR’S CONTRIBUTIONS

BEN, AT, TM, IOF, CC, SR and GT conceptualized the study. BEN, BT, NN‐M and MVS led cost data collection. BT, NN‐M, AT, TM, MVS, IOF, CC and GF led study data collection. BEN led data analysis and RC, NL, GF contributed to data analysis. All authors contributed to data interpretation. BEN, RC, SR and GF wrote the first draft of the manuscript. All authors critically reviewed a revised draft of the manuscript. All authors have read and approved the final manuscript.

## ABBREVIATIONS

3MF three‐monthly facility refills; 3MC three‐monthly CAG ART refills; 6MCD six‐monthly community ART refills; ART Antiretroviral treatment; CAD Community ART distribution; CAG Community adherence group; DSD Differentiated service delivery; HIV human immunodeficiency virus; LMIC low‐ and middle‐income countries; LTFU Loss to follow‐up.

## Supporting information


**Supplement File S1.** Methods for estimating unit costs
