# Supplementary material for: Economic evaluation of differentiated service delivery models for HIV treatment in Lesotho: costs to providers and patients
Source: J Int AIDS Soc. 2021 Apr 10;24(4):e25692. doi: 10.1002/jia2.25692 (PMC8035675; doi:10.1002/jia2.25692)
Supplement: Supplementary file 1 — Supplement File S1. Methods for estimating unit costs [file JIA2-24-e25692-s001.docx]

# **Supplementary file 1: Methods for estimating unit costs**

Facility visits:

Unit costs for facility visits were collected from a sample of three public health facilities, two district hospitals, and one health centre. Unit costs were estimated for four clinic visit events: medical examinations, adherence counselling, blood draws, and drug collection. Unit costs for each event included staff time (both clinical and non-clinical), consumables, overhead, and equipment. Staff time costs were drawn from Lesotho Government MOH health care worker salary scales and then multiplied by the proportion of staff time spent on the evaluated services. Consumables costs were obtained from National Drug Service Organisation (<http://ndso.co.ls/>); overhead costs were sourced from the healthcare facility accounting department, averaged across a six-month period. Equipment costs were collected from furniture and equipment retail stores’ catalogues. Unit cost for facility visits at each facility level were then estimated using the Healthcare Cost Outcomes Model, which allocates fixed and shared costs to facility visits.^17^ A final facility visit cost was then defined as the average cost of a visit for the three types of healthcare facilities.

DSD interactions:

Costs of operationalising and delivering DSD models were obtained from discussions with key study staff involved in implementing the models. Cost data collected for CAG interactions included: direct and support staff costs and level of effort and equipment. Cost of building space utilized was <$0.0001 per visit and was therefore excluded. For CAGs, this specifically included the cost of CAG facilitators, tasked with ensuring the organization of the CAGs. To assign the correct cost per interaction, the total costs over the study period were divided by the number of recorded CAG interactions.

Cost data collected included for community distribution interactions included: direct and support staff costs and level of effort, equipment, and vehicle and vehicle maintenance costs. In the case of community distribution, a driver, nursing assistant, and nursing officer are required for an entire day. The cost of their time for the day was divided amongst the number of patients seen (both ART and non-ART patients) to estimate a cost per ART patient. Fixed vehicle costs were assigned across all community distribution interactions during the study period (distributed across both ART and non-ART patient).

ART and viral loads:

Antiretroviral drugs dispensed over 12 months of follow-up were included. A visit that occurred prior to the 12-month mark, but number of pills dispensed would take the patient beyond the 12-months mark, only the number of days of ART until that 12-month mark were included. The number of days of ART was then multiplied by the price of ART in Lesotho. The number of viral load tests that occurred during the 12 months of follow-up were then multiplied with the cost of a viral load from the local laboratory pricelist.
